# Letter to the Editor regarding study design for diagnostic performance of S-detect in differentiating breast masses

**DOI:** 10.1093/bjr/tqaf109

**Published:** 2025-05-24

**Authors:** Jatin Sridhar Naidu, Hitesh Muthyala

**Affiliations:** Division of Surgery and Interventional Sciences, University College London, London, United Kingdom; UCL Medical School, Faculty of Medical Sciences,, University College London, London, United Kingdom; UCL Medical School, Faculty of Medical Sciences,, University College London, London, United Kingdom

**Keywords:** ultrasound, breast masses, s-detect, computer-aided diagnostics

Dear Editor,

We commend Du et al.^1^ for their recent study on the diagnostic performance of the S-detect computer-aided diagnostic (CAD) system. Their dual-centre, three-arm study design, along with their consideration of the radiologist learning curve, enhances the study’s validity. The authors’ exploration of CAD’s potential in breast ultrasound interpretation is particularly valuable.

However, certain methodological aspects merit further consideration. The exclusion of poor-quality ultrasound images—accounting for approximately 10% of cases in our experience—may have led to an overestimation of diagnostic performance. Additionally, the study’s inclusion of only Chinese participants, a limitation common to many studies in this field^2–5^ reduces its generalizability to other populations. The crossover study design, in which radiologists reported both individually and with S-detect, does not fully mitigate potential learning bias. Moreover, the manual adjustment of scans using S-detect introduces an additional source of bias, as the modification process was not clearly described. While the study acknowledges that CAD may reduce inter-observer variability, this was not quantitatively assessed using kappa statistics, which would have strengthened the analysis.

To improve external applicability, future studies could incorporate broader inclusion criteria and adopt a study design where radiologists assess images with and without S-detect at separate times, blinded to their prior interpretations. Prospective studies incorporating multivariate analyses—including factors such as lesion size, patient age, and tumour type—could provide deeper diagnostic insights.
